# Computational analysis of the mutations in BAP1, PBRM1 and SETD2 genes reveals the impaired molecular processes in renal cell carcinoma

**DOI:** 10.18632/oncotarget.5147

**Published:** 2015-10-07

**Authors:** Francesco Piva, Matteo Giulietti, Giulia Occhipinti, Matteo Santoni, Francesco Massari, Valeria Sotte, Roberto Iacovelli, Luciano Burattini, Daniele Santini, Rodolfo Montironi, Stefano Cascinu, Giovanni Principato

**Affiliations:** ^1^ Department of Specialistic Clinical and Odontostomatological Sciences, Polytechnic University of Marche Region, Ancona, Italy; ^2^ Department of Medical Oncology, AOU Ospedali Riuniti – Polytechnic University of the Marche Region, Ancona, Italy; ^3^ Department of Medical Oncology, University of Verona, Verona, Italy; ^4^ Medical Oncology Unit of Urogenital and Head & Neck Tumors, European Institute of Oncology, Milan, Italy; ^5^ Department of Medical Oncology, Campus Bio-Medico University of Rome, Rome, Italy; ^6^ Pathological Anatomy, Polytechnic University of the Marche Region School of Medicine United Hospitals, Ancona, Italy

**Keywords:** mutations, polymorphisms, predictions, RCC, computational

## Abstract

Clear cell Renal Cell Carcinoma (ccRCC) is due to loss of von Hippel–Lindau (*VHL*) gene and at least one out of three chromatin regulating genes BRCA1-associated protein-1 (*BAP1*), Polybromo-1 (*PBRM1*) and Set domain-containing 2 (SETD2). More than 350, 700 and 500 mutations are known respectively for *BAP1, PBRM1* and *SETD2* genes. Each variation damages these genes with different severity levels. Unfortunately for most of these mutations the molecular effect is unknown, so precluding a severity classification. Moreover, the huge number of these gene mutations does not allow to perform experimental assays for each of them. By bioinformatic tools, we performed predictions of the molecular effects of all mutations lying in *BAP1, PBRM1* and *SETD2* genes. Our results allow to distinguish whether a mutation alters protein function directly or by splicing pattern destruction and how much severely. This classification could be useful to reveal correlation with patients’ outcome, to guide experiments, to select the variations that are worth to be included in translational/association studies, and to direct gene therapies.

## INTRODUCTION

Human Renal Cell Carcinoma (RCC), both sporadic and hereditary forms, comprises different histological subtypes. The first carcinogenic step leading to clear cell RCC (ccRCC) is the inactivation of the von Hippel–Lindau (*VHL*) tumour suppressor gene, mapped on 3p25. In particular, one *VHL* allele is usually damaged by a point mutation, while the other allele is lost owning to a large deletion. This deletion can also remove one allele of the near chromatin regulating genes BRCA1-associated protein-1 (*BAP1*), Polybromo-1 (*PBRM1*) and Set domain-containing 2 (*SETD2*). Subsequently, a second-hit mutation can destroy one of the three remaining alleles, causing loss of heterozygosity and leading to different ccRCC grades and aggressiveness dependent on the gene lost [1].

BAP1 controls cell cycle, growth, response to DNA damage and chromatin architecture by histone H2A deubiquitination. In turn, this makes different target genes promoters accessible to transcription factors. PBRM1, which belongs to PBAF (also SWI/SNF-B) complex, binds acetylated H3 histone to remodel chromatin and control cell cycle. SETD2 trimethylates H3 histone controlling chromatin accessibility of genes regulating cell cycle, apoptosis and DNA double strand break signalling. Different studies have reported a mutation frequency of about 33% for *PBRM1*, 10% for *BAP1* and 12% for *SETD2* in ccRCC [2].

Genomic alterations can cause alterations at different molecular levels. A variation lying in the promoter region could increase or decrease the transcript amount by acting on transcription factor (TF) binding sites. The prediction of these events is a few reliable since the promoter of each gene has an unknown extension, the TFs can bind short and degenerate motifs, the interactions among transcription factors on a promoter are largely unknown. An alteration lying in exons or introns can remove a splicing isoform or create an ectopic one. Naturally, if the tract appeared or disappeared in mRNA is not multiple of 3bp there is a frameshift error and there could be a premature termination codon that gives rise to a truncated protein. A part from the splicing, mutations lying in coding exons can also alter the protein function directly, by changing one or more aminoacids or introducing early stop codons. A variation in 3′UTR can destroy a microRNA binding site and lead to the increase of the related protein, on the contrary the creation of a site can reduce the protein level.

In this study, we predicted, by bioinformatics tools, the effects of all known mutations in *BAP1, PBRM1* and *SETD2* genes and we discussed their potential implications in guiding the management of RCC patients.

## RESULTS

In Table 1 we reported a synthesis of the full results (Supplementary Table 1). Regarding splicing alterations, we adopted the following criteria to interpret prediction results: (i) the weakening of a natural splice was judged as severe if its score was decreased of at least 0.4 or the final score was lesser than 0.4. These events could lead to the skip of the splice site. We considered as mild the small score deviations (for example, from 0.39 to 0.30). (ii) The strengthening of a cryptic splice site was judged as severe if its final score was greater than 0.4 since this could activate the splice site. If the score did not reach 0.4 the event was judged as mild. (iii) The creation of a binding site in an exon for the exonic silencer factor hnRNP A1 was judged as severe because this protein acts as a strong silencer and can cause exon skipping. The creation of binding sites for other exonic silencers was judged as mild. (iv) The loss of a binding site for an exonic enhancer was judged as neutral since pre-mRNAs are rich in these sites (known as redundancy) and not frequently these events alter the splicing. However, we have to remind that there are not yet exact rules to model the splicing process. In fact, up till now, given a new pre-mRNA sequence it is not possible to predict the position of the exon and the alternative splicing isoforms. Therefore we dopted general guidelines deduced by mutational analysis present in the literature. We considered as severe a mutation predicted to be severe according to either NNSPLICE or SpliceAid 2. Analogously for protein effect, we graded as severe a mutation if either PredictSNP or DDIG-in claimed the severity. A mutation was considered as mild for splicing if it was mild for NNSPLICE or for SpliceAid 2 or for both tools and, analogously, for the protein prediction tools. Finally, a mutation that was predicted neutral by both splicing tools was defined neutral, and analogously for the protein tools. If at least one out of the four tools indicated a severity it was reported as “Severe” and if at least one tool predicted a mild effect but there were not severe judgments, it was indicated as “Mild”. Clearly, when all tools did not point out alterations, the mutation was classified as “Neutral”. According to our experience, we made an exception, considering as mild, rather than severe, the few in frame insertions or deletions, because they generally added or deleted one or more aminoacids not changing the reading frame.

**Table 1:** Synthesis of the predictions of the effect of the analyzed mutations

Gene	Mutation type		Severity	Effect on Splicing	Effect on Protein	Summary
***BAP1*(383)**	Missense	174 (45%)	Severe	22	106	115
			Mild	36	0	11
			Neutral	106	68	48
	Nonsense	42 (11%)	Severe	9	42	42
			Mild	10	0	0
			Neutral	23	0	0
	Synonymous	20 (5%)	Severe	3	-	3
			Mild	5	-	5
			Neutral	12	-	12
	Frameshift	99 (26%)	Severe	8	99	99
			Mild	24	0	0
			Neutral	67	0	0
	In frame indels	10 (3%)	Severe	1	0	1
			Mild	0	9	8
			Neutral	9	1	1
	Splicing site	38 (10%)	Severe	32	-	32
			Mild	5	-	5
			Neutral	1	-	1
***PBRM1*(715)**	Missense	234 (33%)	Severe	26	116	129
			Mild	59	0	34
			Neutral	147	118	71
	Nonsense	119 (17%)	Severe	28	119	119
			Mild	20	0	0
			Neutral	71	0	0
	Synonymous	32 (4%)	Severe	3	-	3
			Mild	6	-	6
			Neutral	23	-	23
	Frameshift	248 (35%)	Severe	24	248	248
			Mild	40	0	0
			Neutral	184	0	0
	In frame indels	21 (3%)	Severe	2	0	2
			Mild	4	20	18
			Neutral	15	1	1
	Splicing site	61 (8%)	Severe	50	-	50
			Mild	7	-	7
			Neutral	4	-	4
***SETD2*(511)**	Missense	303 (59%)	Severe	26	138	156
			Mild	43	0	37
			Neutral	234	161	110
	Nonsense	95 (19%)	Severe	9	92	95
			Mild	16	0	0
			Neutral	70	0	0
	Synonymous	40 (8%)	Severe	2	-	2
			Mild	6	-	6
			Neutral	32	-	32
	Frameshift	52 (10%)	Severe	6	50	52
			Mild	6	0	0
			Neutral	40	0	0
	In frame indels	4 (1%)	Severe	0	0	0
			Mild	0	3	3
			Neutral	4	1	1
	Splicing site	17 (3%)	Severe	12	-	12
			Mild	2	-	2
			Neutral	3	-	3

We reported the number of mutations having severe, mild and neutral effects at splicing and at protein levels. “-“ this kind of tool was not applicable at that kind of mutation. In some cases the sum of occurrence of a same group of mutations does not correspond to the total count shown in “Mutation type” column because the tools gave back an error message or the nucleotides added following an insertion, or their exact position, were not known. For example, protein predictions of SETD2 result to be 92, 0, 0 but their sum is not 95.

We collected 383, 715, 511 mutations respectively for *BAP1, PBRM1* and *SETD2* genes, of which the majority were missense and frameshift. All nonsense and frameshift mutations were predicted to be severe. Generally, the severity of these kinds of variations can be proportional to the number of aminoacids altered. As a consequence, if a mutation lies in the C-terminal region of the protein, no serious damages for protein function could be assumed. Instead, for BAP1 (729 AAs) also variations involving the last aminoacids provoke severe alterations. In fact, the AAs from 717 to 722 constitute the nuclear localization signal (NLS), whose modification leads to protein retention in the cytoplasmic compartment [3].

**Figure 1:**
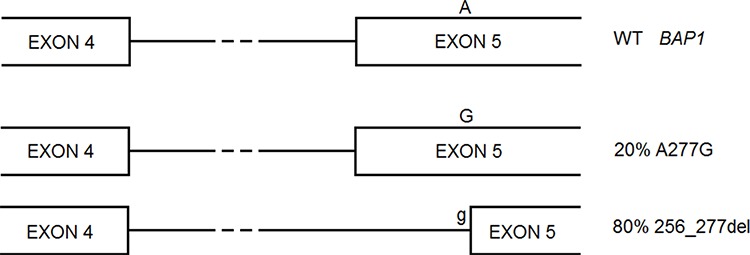
*BAP1* A277G is a germline mutation that gives rise to two alternative splicing forms, one carrying the missense mutation, the other lacking of part of exon 5 and causing frameshift

Our prediction that *BAP1* c.A277G mutation created a 3′ splice site in an exon and so could lead to skip part of the exon is in accord with literature data (Figure 1) [4]. Analogously, our predicted alterations for BAP1 Ser63Cys, Phe81Val, Cys91Trp, Ala95Asp were consistent with enzymatic assays [5]. Also prediction on protein effect for SETD2 Arg1625His is coherent with literature data, which report loss of methyltransferase activity [6].

Missense mutation c.2054 A > T (p.Glu685Val) in *BAP1* exon 16 (−3 position within exon) was assessed to affect splicing rather than protein sequence and structure. In particular, it created a novel 5′ splice site, completely disrupting normal splicing of *BAP1*, by creating both exonic 4nt deletion and retention of neighbouring introns. Besides creation of an alternative 5′ splice site (5′ss), a novel 3′ splice site (3′ss) was created that caused likewise intron retention [7]. These data confirm our predictions for this mutation, that is the disruption of the normal 5′ss, the creation of an alternative 5′ss at the assessed position and, finally, the creation of a 3′ss.

*BAP1* c.438–1G > A mutation was predicted to disrupt a 3′splice site and so to loss or gain nucleotides, potentially leading to frameshift. This could trigger the nonsense mediated decay (NMD) surveillance mechanism that eliminates the potentially dangerous transcript. In this case, immunohistochemical (IHC) data are available and state the protein absence [1]. Also for *PBRM1*, the effect of some splice site mutations (c.1347+1G > T, c.2683+1G > A, c.3773_3779+9del16, c.2472–1G > A, c.3780–18_3780–2del17) that destroyed splice sites and a missense mutation (c.3752T > A Val1251Glu) that creates a 5′ss, the IHC data, claiming protein absence, supported previous hypothesis.

Although many other predictions are consistent with the few IHC data, there are some discrepancies. For example, despite the *BAP1* c.128T > G (Val43Gly), c.283G > C (Ala95Pro) and c.335T > C (Leu112Prp) and *PBRM1* c.236G > T (Arg79Ile) and c.3014T > G (Val1005Gly) missense mutations could alter the splicing and/or change at most only one aminoacid, they even abrogate protein expression according to IHC data. This could be explained by the predicted creation of binding sites for exonic splicing silencer factors, leading to a splicing alteration and to a possible frameshift that, in turn, can trigger the NMD mechanism.

*BAP1* c.21_32del12 (E7_S10delGluLeuGluSer) is an in frame deletion of 4 AAs, that DDIG-in predicted to disrupt protein function, but surprisingly the IHC data show the complete protein absence. As for *BAP1* c.2188T > G (*730Gly), it eliminates the stop codon leading to protein extension instead IHC data was negative. Probably this is due to the appearance of an exonic silencer as predicted by SpliceAid and the probably following NMD priming.

*PBRM1* c.2703delT (Ser902fs*80) and c.3292G > T (Glu1098*) are a frameshift and a nonsense mutations, respectively, that DDIG-in predicted to be deleterious and we expected that they prevented the PBRM1 expression. However, surprisingly, IHC data are positive for these protein variants. We did not find creation or destruction of motifs that regulates the RNA nuclear export.

These data are useful to select high-risk mutations that are worth to be evaluated in clinical studies. Moreover, we suggested which mutations alter molecular mechanisms more severely and should be investigated first at splicing or protein level. The knowledge of these mutations allows to design the targets for recent systems, as the CRISPR/Cas9 repairing system, that specifically edit DNA loci to add or delete base pairs [8].

## DISCUSSION

The Cancer Genome Atlas Research Network supports the fundamental role of oxygen-sensing genes (*VHL*) and chromatin remodeling genes (*PBRM1, BAP1* and *SETD2*) in RCC tumorigenesis [9], highlighting their potential as prognostic biomarkers and/or future therapeutic targets.

However, several issues still need to be elucidated. First, what kind of different mutations damaging these chromatin-remodeling genes can occur, and how do they contribute to RCC development? How severe is a mutation lying in *BAP1, PBRM1* and *SETD2* genes in terms of protein function impairment, being largely unknown the molecular effects of all these genes alterations?

Using the COSMIC and MutDB mutation databases, we identified roughly 380, 700, 500 mutations for *BAP1, PBRM1* and *SETD2* genes, respectively.

Truncating mutations (base substitutions, insertions or deletions) lying in *VHL, BAP1, PBRM1* and *SETD2* can result in frameshift and nonsense mutations, directly determining loss of the corresponding protein or function. In our study, all nonsense and frameshift mutations have been predicted to be severe.

Furthermore, both exonic and intronic mutations (synonymous and missense) have to be considered as potential mechanisms of splicing alterations leading to partially or totally exon skipping or intron retention, causing potential severe effects. As a consequence, splicing alterations could trigger a surveillance mechanism, named NMD, which provokes transcript degradation and protein loss, and resulting in the immunohistochemical lack of protein staining.

Second, can we be confident in a narrow and linear correlation between genomic sequencing data and those of easier access resulting from immunohistochemistry? Although the loss of a protein expression (or secondary to splicing alterations) generally leads to the corresponding absence of positivity to immunohistochemical staining, some discrepancies have been highlighted (i.e. *PBRM1* c.2703delT (Ser902fs*80) frameshift mutation is associated unexpectedly with positive IHC staining), which impede to consider the latter method (less expensive and of easier access) as the standard technique.

Third, are there correlations among *BAP1, PBMR1* and *SETD2* mutated genes? And, is it possible to identify a link between these genes and patients prognosis and/or response to treatments, attributing to them a prognostic or predictive value?

Interestingly, mutations in *PBRM1* and *BAP1* are largely mutually exclusive, while *PBRM1* and *SETD2* mutations frequently co-occur in tumors, cooperating in RCC tumorigenesis [12]. Concerning the prognostic significance of *BAP1, PBRM1* and *SETD2* mutational status, *BAP1* loss correlates with high Fuhrman nuclear grade and mTORC1 activation [1], higher tumor stage, and worst overall survival [13], while *PBRM1* mutations seem to identify a favorable group of ccRCC.

Nevertheless, our study presents several limits. The correct intron removal depends on several variables such as the competition among many enhancing and silencing splicing factors, their proximity to splice sites, the RNA secondary structure and the cellular context. Since a reliable RNA secondary structure prediction lacks and, up till now, the result of a splicing factor competition is impossible to predict, we performed our splicing judgements adopting the best-accepted criteria for bioinformatic analyses. Moreover, protein prediction tools and NNSPLICE, since they have been trained with datasets collecting a limited number of sequences, may suffer of some false negative and false positive discovery rate.

Interestingly, the effect of mutations could involve also less known molecular levels. For example, we assessed that 6, 19 and 47 circular RNAs (circRNAs) rise respectively from *BAP1, PBRM1* and *SETD2* loci, according to circBase (www.circbase.org). Their biogenesis and function, although have to be yet elucidated, could be altered by all kind of mutations [14].

In conclusion, we ranked *BAP1, PBRM1* and *SETD2* mutations based on their predicted effect on splicing and protein function, allowing to identify the damage severity, so as to select high-risk mutations that are worth to be evaluated in clinical trials. Our results provide the basis for an integrated pathological and molecular genetic classification of ccRCC, which takes into account disease-specific panels of genes, such as *VHL, BAP1, PBRM1* and *SETD2*, thereby paving the way for subtype-specific treatments that exploit genetic abnormalities.

## MATERIALS AND METHODS

### Collection of mutations

We extracted mutation data of *BAP1, PBRM1* and *SETD2* from COSMIC (http://cancer.sanger.ac.uk) [15] and MutDB (www.mutdb.org) [16] mutation databases. COSMIC, the Catalogue Of Somatic Mutations In Cancer, is a large collection of human point mutations (insertions, deletions, silent, missense and nonsense mutations) observed in tumour samples and it is manually curated from the scientific literature. MutDB collects both nonsynonymous SNPs and mutations with protein structural information obtained from UniProt.

### Predicition of protein alterations

Many computational tools are already widely employed for the prediction of the effects of mutations on protein function. For the predictions of the missense mutation effects we used PredictSNP [17]. In particular, this resource is a consensus classifier that processes the predictions of MAPP, nsSNPAnalyzer, PANTHER, PhD-SNP, PolyPhen-1, PolyPhen-2, SIFT and SNAP tools to reach better prediction performances than the individual tools and other consensus tools as CONDEL and Meta-SNP do. The mutations were categorized as neutral or dangerous, with the related confidence level ranging from 0 (low) to 1 (high). In order to discriminate disease-causing from neutral frameshifting insertions or deletions (indels) and nonsense variants, that disrupt the protein coding sequence downstream of the mutation, we used DDIG-in (http://sparks-lab.org/ddig) [18]. It is a machine-learning tool that can predict the disease probability, since it is trained on inherited disease-causing mutations from the Human Gene Mutation Database (HGMD) and putatively neutral variants from the 1000 Genomes Project.

### Prediction of splicing alterations

In order to predict if mutations create or destroy 5′ and 3′ splice sites we used NNSPLICE tool [19] that uses an artificial neural network (ANN) algorithm to estimate the splice sites strength. Moreover it was assessed that this resource has good performances in different works [20, 21]. NNSPLICE searches for splice sites in submitted sequence and gives back a score to the detected splice sites ranging from 0 (weak) to 1 (strong). We submitted both WT and mutated sequences to compare the weakening or strengthening of functional splice sites and the creation of new sites. We considered mutations with the score differences higher than 0.2 as splicing altering as adopted by other authors [22]. Detailed interpretation of the consequences of the splice site alteration events was previously reported [23].

To predict if mutations lie in splicing regulatory sequences recognized by splicing factors we used SpliceAid2 tool (http://www.introni.it/spliceaid.html) [24]. This resource uses only experimentally assessed target RNA sequences in humans and therefore reduces the false positive results. The complete list of the splicing enhancer and silencer factors was previously published [25]. SpliceAid2 detects the position of enhancer or silencer elements in sequence submitted by users. Notably, the creation of exonic silencers is usually a severe event that can lead to skip a part or the entire exon whereas its loss could alter the inclusion rate of an alternative exon. The loss or gain of an exonic enhancer is usually tolerated because these motifs are abundant in exons.

### Prediction of mRNA export alterations

The nucleocytoplasmic export of a transcript can be facilitated or obstructed by specific elements, as the human eIF4E Sensitive Element (eIF4E-SE), c-Jun gene Enhancer (CJE), Cytoplasmic Accumulation Region (CAR), Post-Transcriptional Regulatory Element (PRE), Constitutive Transport Element (CTE) and Signal Sequence Coding Region (SSCR). All these and other elements are collected in ExportAid tool [26] that we used to detect if a mutation alters, creates or destroys mRNA export elements. This resource gives back the results as BLAST alignments.

## SUPPLEMENTARY TABLE




