# Supplementary material for: Computational analysis of the mutations in BAP1, PBRM1 and SETD2 genes reveals the impaired molecular processes in renal cell carcinoma
Source: Oncotarget. 2015 Oct 7;6(31):32161–8. doi: 10.18632/oncotarget.5147 (PMC4741666; doi:10.18632/oncotarget.5147)
Supplement: Supplementary file 1 [file oncotarget-06-32161-s001.pdf]

SUPPLEMENTARY TABLE

Supplementary Table S1: We coloured in red the predictions considered as Severe, orange those Mild and not coloured those neutral, a part from in "Summary" column where neutrals are in green. For NNSPLICE column, the formalism "5′(<0.30 —> 0.97)" means that in wt sequence there was not a 5′ splice site or it was weak. The mutations creates a strong 5′ splice site.
The destruction of an existing splicing site or the creation of a new splicing site, detected by a score difference of at least 0.2, were considered as Severe.
The cases with an undefinited score (<0.30) and the other score <0.50 not certainly had a difference >0.2 so we attributed a Mild judgement.
For SpliceAid 2 column, the formalism ";-HuB, +SRp38" means that the mutation destroyes a HuB binding site and creates a binding site for SRp38. According to Material and Methods section, the creation of a binding site for a silencer in an exon was considered Mild because could lead to partial or total exon skipping. If the predicted silencer was hnRNP A1, we assigned a Severe judgement because this is the strongest exonic silencer known.
PredictSNP and DDIG-in gave only two judgments, of which one neutral and one severe.
"N/A" means that the nucleotides added following an insertion or the exact positions of a mutation were not known so it was impossible to perform predictions.
"N/R" means that the tools gave back an error message.
"–" means that the prediction tool was not applicable to that type of mutation. For example, synonym or intronic mutations cannot be processed by PredictSNP or DDIG, that are designed for aminoacidic alterations.
In the "Summary" column the judgement was red if at least one out of four prediction tools gave a Severe evaluation. Green colour means that all tools gave a Neutral assessment and orange in the remaining cases.
The "PredictSNP details" sheet shows the predictions of all tools used by PredictSNP to reach the final verdict.
